# Tetranucleotide usage in mycobacteriophage genomes: alignment-free methods to cluster phage and infer evolutionary relationships

**DOI:** 10.1186/1471-2105-16-S2-A7

**Published:** 2015-01-28

**Authors:** Benjamin Siranosian, Emma Herold, Edward Williams, Chen Ye, Christopher de Graffenried

**Affiliations:** 1 Center for Computational Molecular Biology, Brown University, Providence, RI, USA; 2 Division of Biology and Medicine, Brown University, Providence, RI, USA; 3 Department of Molecular Microbiology and Immunology, Brown University, Providence, RI, USA

## Background

The genomic sequences of phages isolated on mycobacterial hosts are diverse, mosaic and often share little nucleotide similarity. However, about 30 unique types have been isolated, allowing most phage to be grouped into clusters and further into subclusters [1]. Many tools for the analysis of mycobacteriophage genomes depend on sequence alignment or knowledge of gene content. These methods are computationally expensive, can require significant manual input (for example, gene annotation) and can be ineffective for significantly diverged sequences [2]. We evaluated tetranucleotide usage in mycobacteriophages as an alternative to alignment-based methods for genome analysis.

## Description

**Figure 1:**
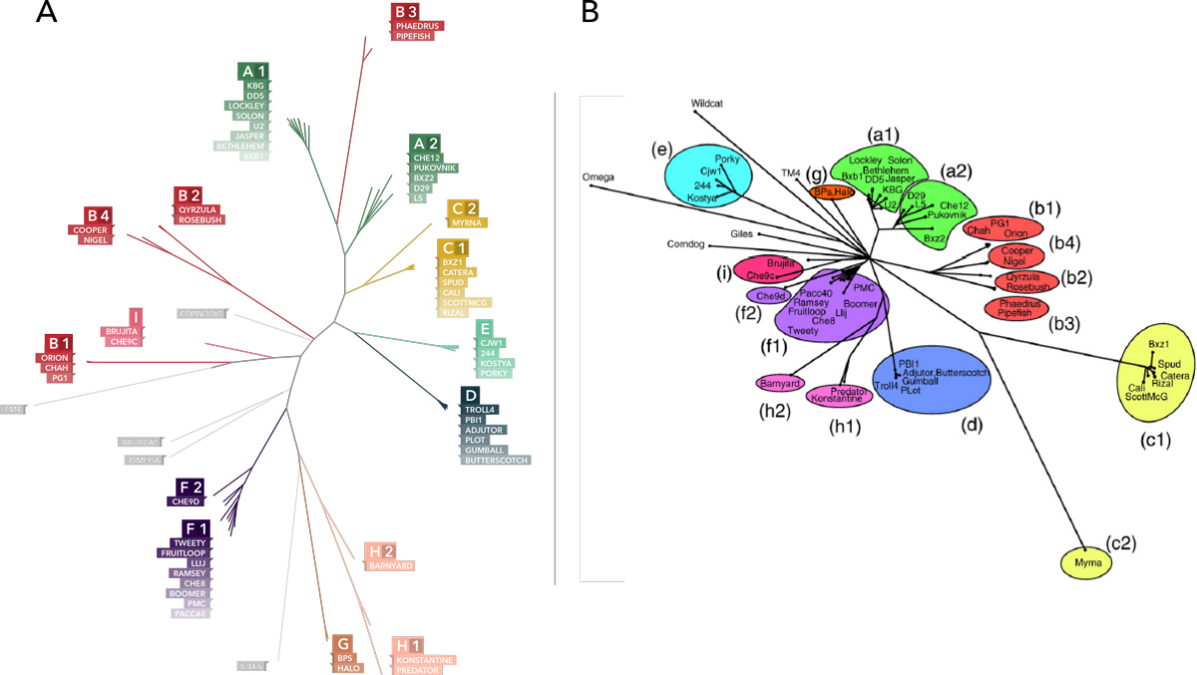
**A) Neighbor joining tree constructed from tetranucleotide usage deviation distances and B) tree from **[4]** constructed from predicted protein products in a subset of sequenced mycobacteriophages**. Our method accurately places phage in a monophyletic clade with members of the same subcluster and often reconstructs relationships between subclusters. In some cases, a subcluster is not placed with other members of the cluster because of significant and conserved differences in tetranucleotide usage, such as overrepresentation of the 4-mer 'GATC' in cluster B3 genomes.

We computed tetranucleotide usage deviation, the ratio of observed counts of 4-mers in a genome to the expected count under a null model [3]. Tetranucleotide usage deviation is comparable for members of the same phage subcluster and distinct between subclusters. Neighbor joining phylogenetic trees were constructed on pairwise Euclidean distances between all genomes in the mycobacteriophage database. In almost every case, phage were placed in a monophyletic clade with members of the same subcluster. With few exceptions, trees computed from tetranucleotide usage deviation accurately reconstruct trees based on gene content for a subset of the mycobacteriophage population (Figure 1). We also evaluated the possibility of assigning clusters to unknown phage based on tetranucleotide usage deviation. Under a simple nearest neighbor classifier, cluster assignments were recovered at a frequency greater than 98%. In addition, we looked for evidence of horizontal gene transfer by using tetranucleotide difference index, a measure of the deviation in tetranucleotide usage from the genomic mean in a sliding window across the genome [3]. Tetranucleotide difference index plots showed a strong spike at the end of cluster L mycobacteriophages, which could indicate horizontal gene transfer in the region.

## Conclusions

Genome analysis based on tetranucleotide usage shows promise for evaluating host-parasite coevolution and gene exchange within the mycobacteriophage population. These methods are computationally inexpensive and independent of gene annotation, making them optimal candidates for further research aimed at clustering phage and determining evolutionary relationships. Code for genome analysis and data used in this project are freely available at https://github.com/bsiranosian/tango_final.
